# Phosphoinositides in Retinal Function and Disease

**DOI:** 10.3390/cells9040866

**Published:** 2020-04-02

**Authors:** Theodore G. Wensel

**Affiliations:** Verna and Marrs McLean Department of Biochemistry and Molecular Biology, Baylor College of Medicine, Houston, TX 77030, USA; twensel@bcm.edu

**Keywords:** phosphoinositides, retinal lipids, membrane trafficking

## Abstract

Phosphatidylinositol and its phosphorylated derivatives, the phosphoinositides, play many important roles in all eukaryotic cells. These include modulation of physical properties of membranes, activation or inhibition of membrane-associated proteins, recruitment of peripheral membrane proteins that act as effectors, and control of membrane trafficking. They also serve as precursors for important second messengers, inositol (1,4,5) trisphosphate and diacylglycerol. Animal models and human diseases involving defects in phosphoinositide regulatory pathways have revealed their importance for function in the mammalian retina and retinal pigmented epithelium. New technologies for localizing, measuring and genetically manipulating them are revealing new information about their importance for the function and health of the vertebrate retina.

## 1. Introduction

**Figure 1:**
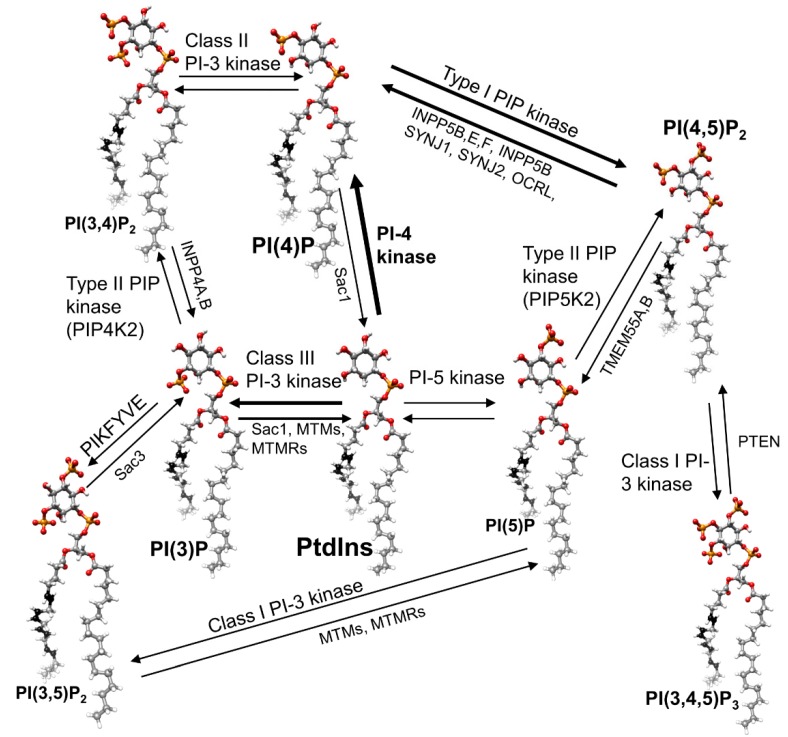
Structures of the cellular phosphoinositides and enzymes responsible for their synthesis and interconversion. Phosphoinositides (PI) species are shown with the acyl chains most commonly found on phosphatidylinositol, which is the starting point for all the others, arachidonic acid (20:4) and stearic acid (C18). Relative font sizes correlate with relative abundance.

Phosphoinositides are membrane phospholipids with the six-member cyclic polyol *myo*-inositol, (CHOH)_6_, or O-phosphorylated forms of inositol, as their headgroup (Figure 1). The phosphorylated forms are of low abundance in eukaryotic cells of all types, generally comprising 1% or less of total phospholipid. Nevertheless, they play critical roles in cellular regulation, and defects in their synthesis and regulation lead to devastating diseases [1,2,3,4,5,6]. The retina is clearly no exception to this generality, but surprisingly few details have been worked out in what is arguably one of the most extensively studied tissues in our bodies, about the regulation and regulatory roles of retinal phosphoinositides. Recent technological advances make it possible to make substantial advances in this field in the next few years.

## 2. Chemical Structures of Phosphoinositides

Phosphoinositides (abbreviated here as “PI”) include phosphatidylinositol (PtdIns), in which the 1′-position of the inositol ring is attached via a phosphodiester bond to the *sn*-3 position of (1, 2) diacyl glycerol, and derivatives of PtdIns with one, two, or three phosphates attached in various combinations to the 4′, 5′ or 3′ hydroxyls of PtdIns. Including PtdIns, a total of eight different PI head groups are commonly found in eukaryotic cells (Figure 1) [4,5].

## 3. Phosphatidylinositol Content in the Retina and RPE

PtdIns is a relatively substantial component of the membranes of most cells in metazoans, with mole fractions ranging from ~4%–20% of total phospholipid [4]. Early reports stated that retina phospholipids contained 4.4%–6.4% PtdIns across six different mammalian species [7,8,9]. Interestingly, rod outer segment (ROS) membranes were found to have much lower levels, 1.5% to 2.5% in bovine and 2.1% in frog [10,11,12,13]. Retinal pigment epithelial cells (RPE), in contrast, have higher levels of PtdIns, at 6.5% of total phospholipid [14], while ER membranes isolated from bovine retinas contain 9.6% PtdIns [10]. The general picture that emerges is that total PtdIns content in total retinal membranes outside of outer segments and that of RPE are more or less “normal” as compared to other tissues and cell types, consistent with the notion that widespread roles of PI are likely to be conserved in many retinal cell types. In contrast, ROS have an unusually low PtdIns content, suggesting different roles for this lipid class in that organelle. This idea is consistent with findings discussed below, indicating different lipid compositions in plasma membranes as compared to ciliary membranes. 

## 4. Content of Minor Phosphoinositides in the Retina and RPE

As a negatively charged lipid, which typically contains one polyunsaturated side chain fatty acid, such as arachidonic acid [8], PtdIns has a major impact on the physical properties of the membrane domains containing it. In contrast, the phosphorylated forms are much less abundant, and exert their influence on cell physiology largely through interactions with proteins with high-affinity and high-specificity PI-binding domains [15,16,17]. There have been few measurements of phosphorylated PI in retina or cells isolated from retina, although there have been several papers addressing the turnover of PtdIns and other PI, or activity of enzymes involved in PI metabolism [18,19,20,21]. The likely reason for the absence of information on PI levels is their low abundance and the lack of sensitivity provided by conventional methods for lipid analysis. More sensitive techniques have been developed recently, including one based on recombinant phosphoinositide-binding domains fused to an epitope tag, allowing sensitive detection by enzyme-linked immunosorbent assays (ELISAs) and measurement of chemiluminescence [22]. This technique was used to quantify PI(3)P and PI(3,4,5)P_3_ in preparations of rod cells that contain both outer segments and fragments of the inner segments and demonstrated levels of PI(3)P at 0.0035 mol% of total phospholipid under illumination conditions that yielded the highest levels of that lipid, and at least 10-fold lower (i.e., undetectable) levels of PI(3,4,5)P_3_. PI(4)P and PI(4,5)P_2_ are, in general, found at much higher levels than the 3-phosphorylated PI, but even those appear to be present in rods at very low levels, which are only about 10-fold higher than the levels of PI(3)P, i.e., on the order of 0.04 mol% (He and Wensel, unpublished observations).

### Comparison to Other Tissues and Cell Types

These numbers are comparable to those found in other eukaryotic cells, reported as PtdIns3P, 0.002% of phospholipid mass; PtdIns4P, 0.05%; PtdIns5P, 0.002%; PtdIns(4,5)P, 0.05%; PtdIns(3,4)P, 0.0001%; PtdIns(3,5)P, 0.0001% [17], see also references in [23]. A more recent mass spectrometry study reported PI(3,4,5)3 levels as 50-fold or more lower than those of the more common PIs in mammalian U87MG cells [24]. An interesting observation derived from measurements of PI levels in cultured cells is that, generally, they do not change greatly upon activation with extracellular stimuli; for the more common forms, PtdIns, PI(4)P, PI(4,5)P_2_ and PI(3)P, the change is generally less than 30% [17]. The implication, for PI(4,5)P_2_, which is rapidly degraded to form InsP_3_ and diacylglycerol upon activation of phospholipase C isozymes by G-protein-coupled receptors or growth factor receptors [25,26], is that homeostatic mechanisms are in place to regenerate rapidly the pools of both PI(4,5)P_2_ and its precursor PI(4)P upon PLC activation. In contrast, PI(3,4,5)P_3_ level changes due to receptor stimulation are generally too low to have a major impact on levels of its precursor, PI(4,5)P_2_.

## 5. Importance of PI Generally

Despite their low abundance, phosphoinositides play major roles in regulation of cell signaling and membrane dynamics, and are essential for control of a wide range of processes including development, proliferation and differentiation, membrane excitability, exocytosis, phagocytosis, cell motility, and detection of extracellular signals [4]. These functional roles are primarily mediated by a plethora of enzymes, scaffold proteins and complex-nucleating proteins containing phosphoinositide-binding domains of high affinity and high specificity. Genetic defects in the enzymes responsible for their regulation are, in most cases, lethal at the embryo stage, except for some cases of redundancy (i.e., more than one gene encoding enzymes or PI-binding proteins with similar activities). Cell-type specific deletion of these enzymes leads to more specific pathologies, e.g., neurodegeneration in the case of the type III PI-3 kinase, Vps34 (also known as PIK3C3) [27,28]. Some of these enzymes, such as type I PI-3 kinases, can act as oncogenes when mutations disrupt their regulation, and are considered prime targets for cancer chemotherapy, whereas others, such as the phosphoinositide phosphatase, PTEN, are considered tumor suppressor genes [29]; these therapeutic targets are present in the retina, where their roles in retinal function, and any effects of drugs targeting them are unknown.

### Importance of Phosphoinositides for Dynamics and Functions of Membranes in Retina and RPE

Among the most important roles of phosphoinositides and their protein effectors are those mediating intracellular membrane traffic, by directing membrane proteins and lipids from one compartment to another in response to cellular needs and changing environments. For example, PI(3)P is found in early and recycling endosomes, and is important for recruiting key proteins that regulate trafficking to these compartments [30]. It is also important for autophagy, a survival-promoting pathway leading to the lysosomal degradation of organelles [31]. PI(4)P is enriched in the Golgi apparatus, and is thought to be important for membrane trafficking through the Golgi compartments and from the Golgi to the plasma membrane and other subcellular compartments [32], likely including disk membranes. PI(4,5)P_2_ plays a key role in clathrin-mediated endocytosis, and serves to direct many effector proteins to the plasma membrane where it is primarily found [33].

PI(4,5)P_2_ and other phosphoinositides directly regulate the activity of ion channels, transporters and enzymes in membranes [34,35]. In addition, PI(4,5)P_2_ serves as the substrate for phosphoinositide-specific phospholipase C, leading to production of the important second messengers, inositol (1,4,5) trisphosphate (InsP_3_) and diacylglycerol [25,26,36,37]. Phosphoinositides are also critically important for regulating interactions between membranes and cytoskeletal elements [38,39]. Changes in these interactions are critical for cell growth and mobility, and for remodeling of intracellular structures. They may well play a role in cytoskeleton-dependent disk morphogenesis in rods.

## 6. Membrane Trafficking in Retina and RPE

Every aspect of retinal biology depends heavily on the correct organization and composition of highly specialized membranes, from the unique disc membranes of the photoreceptor sensory cilia, to the ribbon synapses of rods, cones, and bipolar cells, to the apical processes of RPE (retinal pigmented epithelium) cells, uniquely tuned to the detection and engulfment of shed outer segment fragments. The formation, maintenance, and functions of these membranes rely heavily on the phosphorylated phosphoinositides [2,3,4,40]. Despite the intense interest in phosphoinositide research in recent decades, surprisingly little is known about their regulation and functional roles in the retina, although it is known that disruption of phosphoinositide regulation can lead to blindness in human patients and animal models [41,42,43,44,45,46,47,48]. The membranes and pathways they regulate are known to be essential for the function and health of the retina as well as for disease processes and cellular responses to disease states.

One of the reasons for the dearth of knowledge has been a lack of tools for studying these very low-abundance lipids within the multiple cell types of the retina and the adjacent retinal pigment epithelium. Recently, tools developed by the broader PI field have begun to be applied to the unique challenges and opportunities posed by the retina [22,49]. Doing so will have enormous impact on our understanding of the cell biology of the retina and its disruption in disease. This can help to inform the design and optimization of therapies aimed at treating and preventing retinal dysfunction and degeneration.

## 7. Features of the Retina that Make It Ideal for Studies of Phosphoinositide Regulation In Vivo

The field of phosphoinositide regulation has long been dominated by studies in cultured immortalized cell lines, giving rise to a critical need for elucidation of their physiological regulation in terminally differentiated neurons. For this reason, in vivo studies have broad significance for this field. There are several features of the retina that make it particularly amenable to studies of phosphoinositide regulation in vivo. These include the ability to assay both structure and function non-invasively, the ease of making cell-type-specific knockouts, the ability to isolate rod cells for either biochemical analysis or ultrastructure determination, an extensive understanding of biochemistry and cell biology, especially of rods, which exceeds that of any of other vertebrate neurons, and a wealth of knowledge of RPE cell biology.

## 8. Importance of Phosphoinositides for Membrane Trafficking in Retina

Despite years of study, no convincing evidence has accumulated for an important role for phosphoinositides in the phototransduction cascade. In contrast, a steady stream of evidence supports a central role for these lipids in membrane trafficking and sorting in all mammalian cell types, just as a critical role in photoreceptors for membrane sorting and trafficking has long been established [3,50,51]. A review article in 2011 covered advances in understanding the roles of phosphoinositides in photoreceptors [6]. It is highly likely that events such as endocytosis and exocytosis, endosomal sorting, membrane budding, post-Golgi vesicle trafficking, and disk morphogenesis all depend on phosphoinositide dynamics, and published reports support a role for PI(3)P and PI(4,5)P_2_ in rhodopsin trafficking [52,53]. For example, PI(4,5)P_2_-binding proteins, ezrin and moesin, were reported to colocalize with Rac1 and Rab8 on rhodopsin transport carrier vesicles at the site of their fusion with the plasma membrane. A recent report suggesting the involvement of actin-nucleating proteins Arp2/Arp3 in basal disc extension [54] potentially implicates local pools or PI(4,5)P_2_, which are known to be critical for their function [55,56].

In cone photoreceptors, ablation of a type I PI-3 kinase leads to enhanced sensitivity to light damage [57]. Mutations in the phosphoinositide phosphatase, synaptojanin 1 lead to defects in synaptic vesicle trafficking in cone cells [44].

## 9. Retinal Cilia and Phosphoinositides

Phosphoinositides, and especially PI(4,5)P_2_ and PI(4)P, have been proposed to play important roles in assembly, disassembly, and regulation of primary cilia [52,58,59,60,61,62]. The lipid content of cilia is different from that of the plasma membrane, and a membrane diffusion barrier surrounding the cilium has been demonstrated (see [63] for a review). Two PI-5-phosphatases, INPP5E and OCRL, essential for cilium function have been reported to be localized to the cilium [41,64,65,66]

### Phosphoinositides and the BBSome

The BBSome, a heterooctameric protein complex involved in ciliary trafficking, whose defects lead to the blinding ciliopathy, Bardet–Biedl syndrome, binds to membranes, and shows a preference for acidic lipids, including phosphoinositides [67,68], and isolated BBS5 which contains pleckstrin homology domains, binds to phosphoinositides, especially PI(3)P [68]. More recently, it was shown that a core BBSome complex containing BBS 1, 4, 5, 8, 9 and 18 and a smaller sub-complex lacking BBS1 and BBS5 bind phosphoinositides with similar specificities. A caveat for these studies is that the commercial “PIP strips” used have local surface densities of phosphoinositides that far exceed anything found under physiological conditions, so further investigation of phosphoinositide-binding of the BBSome and its sub-complexes is warranted.

## 10. Autophagy and other Stress Responses Involving Redirection of Membrane Traffic and Phosphoinositides

It has been reported that light exposure induces elements of the autophagy pathway in rods and that autophagy plays an important role in photoreceptor homeostasis [69,70,71,72,73]. This pathway has been suggested to be a neuroprotective one that forestalls apoptosis under conditions of stress [69,74]. This process may be part of a more general neuroprotective response involving re-direction of membrane traffic and phosphoinositides. As discussed below, Synaptojanin-1 has been implicated in autophagy in zebrafish cones [75].

## 11. Evidence for Effects of Light on Phosphoinositide Metabolism

A number of early reports in the 1980s suggested that light had measurable impacts on PI metabolism in photoreceptors or the retina generally. Based on measurement of ^32^Pi incorporated into PtdIns by metabolic labeling, it was reported that exposure of isolated frog retina to light decreased levels of PIP_2_ by 14% after 5 s and 37% after 15 s, while levels of PI(4)P, PtdIns and other acidic lipids remained essentially constant [76]. Subsequent publications from the same group reported increased levels of ^3^H inositol and ^32^P into phosphoinositides upon illumination [77]. They also reported PI(4,5)P_2_-specific phospholipase C (PLC) activity in frog photoreceptors, and PLC immunoreactivity in bovine rod outer segments [78,79], as well as PI-kinase and PIP-kinase activities in frog ROS [19]. Another group found that exposure of rat retinas to light led to decreased staining of rod outer segments with anti-PI(4,5)P_2_ antibodies [80,81]. The caveats of those experiments are that it is known that physical properties of outer segment membranes and their protein composition are altered by bright light exposure, and that it is difficult to establish the specificity of such antibody staining. In addition to light, reports on regulation of photoreceptor PLC by Ca^2+^ [82] or by subunits of the phototransduction G protein, transducin [83], were published, also suggesting a possible influence of light exposure, which is known to control levels of Ca^2+^ and active and inactive forms of transducin subunits.

In contrast, another group [84] reported that bovine rod outer segments have very little PIP kinase activity as compared to the rest of the retina (consistent with the previously reported low level of PI(4,5)P_2_ in outer segment membranes), and that light adaptation had no measurable effect on phosphoinositide metabolism as compared to in vivo dark adaptation. Yet another group, using metabolic labeling with [^3^H]inositol, reported that light led to decreases in PIP_2_ levels without generation of InsP_3_, suggesting light-dependent activity of a phosphatase rather than of phospholipase C [85]. In vitro studies demonstrated that the presence of PI(4,5)P_2_ in membranes could affect the activity of components of the phototransduction cascade [86,87], including the cGMP-gated cation channel and the cGMP phosphodiesterase-transducin complex. The physiological relevance of effects of PI(4,5)P_2_ on phototransduction or of effects of light on PI(4,5)P_2_ metabolism remains untested, and it seems to be possible to explain the entire time-course of rod light responses without invoking any participation by phosphoinositide metabolism [88]. Antibodies specific for isoforms of phospholipase C or G_αq/11_ PLC-coupled subunits revealed the presence of PLCβ4 and G_αq/11_ in rod outer segments, and G_αq_ and other PKC isoforms elsewhere in the retina [89]. The functional roles of these proteins in outer segments are not known. There have been suggestions that slower effects of prolonged light exposure, such as arrestin translocation from the inner to outer segments of rod cells, may be mediated by a phospholipase C cascade [90].

### Light Regulation of PI-3 Kinase

Interest has turned toward Type I PI-3 kinase and PI(3,4,5)P_3,_ with reports of effects of light on the activity of this enzyme in bovine rod outer segments [91,92], which were reported to be mediated by light-stimulated activation and tyrosine phosphorylation of the insulin receptor [93,94,95]. Deletion of the p85α regulatory subunit of Type I PI-3 kinase in cone cells resulted in progressive degeneration of cones, without observable effects on rod survival [57]. Likewise, cone-specific inactivation of the gene encoding the p110α catalytic subunit also resulted in defects in cone survival [96].

In contrast, Type I PI-3 kinase and its product, PI(3,4,5)P_3_, seem to be much less important for rod function. Rod-specific ablation of the p85α gene using two different rod-specific Cre transgenes yielded no obvious defects in retinal morphology or rod cell survival [22,97], although modest effects on kinetics of light response recovery and arrestin translocation were reported in one case [97]. Quantitative analysis of PI(3,4,5)P_3_ levels in rods isolated from dark-adapted or light-adapted retinas revealed levels of this phosphoinositide of more than one order magnitude lower than those found for PI(3)P in the light, or at least two orders of magnitude lower than light-stimulated levels of PI(4,5)P_2_ [22].

## 12. PI(4,5)P_2_ and Phospholipase C in Intrinsically Photosensitive Ganglion Cells

In addition to image-forming light detection mediated by rods and cones, the vertebrate retina also contains intrinsically photosensitive retinal ganglion cells. These contain phototransduction cascades reminiscent of that found in invertebrate rhabdomeric photoreceptors [98,99,100,101,102,103,104]. Light activates melanopsin, encoded by the *Opn4* gene, a visual pigment which is more closely related to invertebrate opsins than to vertebrate opsin [105]. In M1-type ganglion cells, melanopsin photoisomerization leads to activation of a G_αq/11/14_ class G-protein, which activates the phosphoinositide-specific phospholipase C isoform, PLCβ4; the phospholipase presumably acts on PI(4,5)P_2_ as in other cell types, including *Drosophila* photoreceptors, which contain a homologous phospholipase, to produce diacylglycerol and InsP_3_. PI(4,5)P_2_ hydrolysis, in turn, leads to the activation of the cation channels TRPC6 and TRPC7 [104]. In M4 ganglion cells, a different phototransduction cascade involving cyclic nucleotides and cyclic nucleotide-regulated HCN channels, whereas in M2 ganglion cells, both of these cascades operate [102].

## 13. Studies of PI Metabolism in the RPE

A variety of extracellular stimuli acting on tyrosine kinase-associated receptors or G protein-coupled-receptors have been reported to stimulate release of inositol phosphates in cultured RPE cells, presumably derived from PLC action of PI(4,5)P_2_, on a timescale of tens of minutes; effective stimuli included fetal bovine serum, agonists for muscarinic, histamine, and serotonin, peptides, including bradykinin, arginine vasopressin, bombesin and oxytocin, [106,107,108,109]. The physiological relevance of these observations was not explored, but an in vivo study using frogs demonstrated dramatic acceleration of inositol phosphate release, especially of InsP_3_, following stimulation by light [21]. Acutely isolated rat RPE cells were reported to release InsP_3_ in response to induction of phagocytosis by addition of isolated rod outer segments [110]; this InsP_3_ release was not observed in cells from Royal College of Surgeon (RCS) rats, which have a defect in OS phagocytosis due to a deficiency in the receptor tyrosine kinase, MERTK [111]. As in many other cell types, insulin has been reported to stimulate activity of Type I PI-3 kinase to produce PI(3,4,5)P_3_ [95,112,113]. Responses to hypoxia [112,114,115] and elevated glucose [116,117] are also reported to involve this pathway in RPE.

A number of important processes in RPE are known to rely on phosphoinositides, but how they are regulated in these cells is not well understood. Phagocytosis, autophagy, endocytosis and endosome processing, establishment of epithelial cell polarity and extension of microvilli membranes are all known to critically depend on phosphoinositides. For example, both autophagy and phagocytosis involve the recruitment of the ubiquitin-like protein, LC3 [118,119,120], whose recruitment to membranes depends on PI(3)P. Phagocytosis is also thought to require PI(4,5)P_2_ and lysosomal fusion may involve other phosphoinositides such as PI(3,5)P_2_ and PI(5)P [22,121,122,123,124,125].

## 14. Phosphoinositide Kinases and Phosphatase

In mammals, there are 47 genes encoding 19 PI-kinase and PIP-kinases and 28 PIP phosphatases [126]. 

### 14.1. Kinases

The kinase isoforms are divided into three major families: PI 4-kinases (PI4Ks), the PI 3-kinases (PI3Ks), and PIP (PIP) kinases (PIPKs) [127]. The nomenclature for these is a bit confusing, as some enzymes termed “PI-kinases” actually act primarily as PIP kinases. For example, Type I PI-3 kinases primarily use PI(4,5)P_2_ as their substrate to produce PI(3,4,5)P_3_, and there is little evidence for a substantial portion of cellular PI(3)P being formed by these enzymes. When the Type III PI-3 kinase, Vps34, was knocked out in mouse rod cells and phosphoinositide levels measured, the results suggested complete ablation of PI(3)P despite the presence of the Type I enzyme [22]. Several of these have been reported to be expressed in retina or RPE at the level of protein, mRNA or enzyme activity and most, if not all, are likely present at some level; however, as far as their functions, only the Type I and Type III PI-3 kinase have been studied using gene knockouts in the retina and RPE [22,49,57,128]. Global knockouts have been produced for the α, β, and γ isoforms of Type I PIP-kinases, which are the major source of PI(4,5)P_2_ in most cells [16]. Of these, the γ isoform seems to have the highest expression in the retina [129] and in other neurons [130,131] and leads to severe neuronal phenotypes and early postnatal mortality when knocked out [132]. Type II PIP-kinases have been observed in retina and are reported to be regulated by tyrosine phosphorylation [133]. The proteins encoded by the mouse genes, Pip4k2a, Pip4k2b, Pip4k2c, Pip5k1a, and Pip5k1c were all observed in a proteomic study of mouse retina [134].

### 14.2. Phosphatases

The phosphoinositide phosphatases encoded by Inpp1, Inpp4a, Inpp4b, Inpp5e, Mtmr2 and Synj1 (synaptojanin-1) have also been detected in retina by proteomics [134]. As noted below, defects in synaptojanin-1 and INPP5E are associated with retinal defects, as is the phosphoinositide 5-phosphatase, OCRL (oculocerebrorenal syndrome of Lowe) [135,136]. Among the other enzymes detected, almost none were enriched in the rod outer segment fraction as compared to the rest of the retina, consistent with the relatively low PI content of that organelle. One exception was Pip4k2c, the PI(5)P-4-kinase Type II γ isoform. Inactivation of the mouse *Pip4kc2* gene was found to lead to hyperactivation of the immune system, but the retinal phenotype was not examined [137].

## 15. Retinal Phenotypes of Genetic Defects in Genes Related to Phosphoinositide Metabolism and Signaling

Rods and cones have a very high rate of metabolism and biosynthesis of membrane components, due to the energetic demands of the phototransduction cascade and the daily shedding of ~10% of the disk membranes (based on observation of a rate of 9%–13% in the rhesus monkey [138], which have to be engulfed and recycled by the RPE. As highly polarized cells, their function and health depend critically on efficient and accurate transport of the correct proteins and lipids to the correct compartments. A host of human blinding diseases have been linked to defects in membrane transport and sorting [139,140]. In RPE cells, massive amounts of membrane traffic are associated with their role as professional phagophores. Defects in RPE phagocytosis, as in MERTK deficiency [111,141] and Bestrophin deficiency [142,143,144,145], cause retinal degeneration in humans and animal models and have been proposed to play a role in age-related macular degeneration [146].

### 15.1. Phosphatases and Inherited Retinopathies

Inherited defects in the phosphoinositide phosphatase, INPP5E, are associated with the multi-syndromic ciliopathies, Joubert syndrome and Bardet–Biedl syndrome [147,148,149,150], and with retinal degeneration. The substrates for this phosphatase are PI(4,5)P_2_ and PI(3,4,5)P_3_, and its critical role in the cilia suggests that regulating levels of PI(4,5)P_2_ may be important in ciliogenesis and cilium stability.

Synaptojanins are phosphoinositide phosphatases associated with synaptic function [151,152,153], including vesicle uncoating and endocytosis. Synaptojanin-1 deficiency causes severe cone defects in zebrafish and has been implicated in the regulation of autophagy in cones [45,46,75].

Defects in another phosphoinositide phosphatase, OCRL, are associated with a severe ciliopathy, oculocerebrorenal syndrome of Lowe [136]. Symptoms of this disease include glaucoma, a disease of the retina.

### 15.2. PI3NM3

The phosphatidyl inositol transfer protein PIT3NM3 (aka CCL118) is the mammalian homologue of the *Drosophila* retinal degeneration gene, *rdgB*, and defects in it are associated with autosomal dominant cone-rod dystrophy CORD5 [154,155,156,157,158,159]. The protein has also been implicated in cancer metastasis [160]. In zebrafish, the Class I phosphatidylinositol transfer protein β isoform, Pitpnb, is essential for biogenesis and maintenance of double cones [161].

### 15.3. PI Binding Proteins

Proteins with phosphoinositide-binding domains have also been associated with retinal disease. These include the product of the *tub/TUB* gene [162,163] and a related protein, TULP1 [47,164,165,166]. Tub was discovered as the protein encoded by a gene whose mutation in *tubby* mice causes obesity, deafness and blindness. The widely expressed members of the tubby family, including TULP1-TULP4, have a characteristic carboxyl-terminal tubby domain consisting of an alpha helix surrounded by a beta barrel which has been shown to bind to specific phosphoinositides [167]. Both Tub and Tulp1 are expressed in the retina, and TULP1 mutations cause retinitis pigmentosa [47,168].

## 16. Conclusions

While the overall field of phosphoinositide regulation and signaling has made dramatic advances in recent years, our understanding of specific regulatory pathways in specific cell types of the retina remains limited. The emergence of new technologies for cell-type-specific gene manipulation, along with imaging techniques for subcellular localization of phosphoinositides, their regulatory enzymes and their effector proteins, and the development of more sensitive methods for measuring PI levels, bodes well for rapid progress in understanding the roles of retinal phosphoinositides in the coming years.
